# NK-Cell-Mediated Targeting of Various Solid Tumors Using a B7-H3 Tri-Specific Killer Engager In Vitro and In Vivo

**DOI:** 10.3390/cancers12092659

**Published:** 2020-09-18

**Authors:** Daniel A. Vallera, Soldano Ferrone, Behiye Kodal, Peter Hinderlie, Laura Bendzick, Brianna Ettestad, Caroline Hallstrom, Nicholas A. Zorko, Arpit Rao, Naomi Fujioka, Charles J. Ryan, Melissa A. Geller, Jeffrey S. Miller, Martin Felices

**Affiliations:** 1 Masonic Cancer Center, Laboratory of Molecular Cancer Therapeutics, Department of Radiation Oncology, University of Minnesota, Minneapolis, MN 55455, USA; 2 Massachusetts General Hospital, Harvard Medical School, Boston, MA 02115, USA; sferrone@mgh.harvard.edu; 3 Department of Medicine, Division of Hematology, Oncology, and Transplantation, University of Minnesota, Minneapolis, MN 55455, USA; KODA0020@umn.edu (B.K.); phoward@umn.edu (P.H.); ettes017@d.umn.edu (B.E.); challstr@umn.edu (C.H.); zorko004@umn.edu (N.A.Z.); raoa@umn.edu (A.R.); fujio002@umn.edu (N.F.); ryanc@umn.edu (C.J.R.); mille011@umn.edu (J.S.M.); 4 Department of Obstetrics, Gynecology and Women’s Health, Division of Gynecologic Oncology, University of Minnesota, Minneapolis, MN 55455, USA; green711@umn.edu (L.B.); gelle005@umn.edu (M.A.G.)

**Keywords:** NK cells, ADCC, IL-15, bispecific antibodies, carcinoma, innate immunotherapy, TriKEs

## Abstract

**Simple Summary:**

B7-H3 costimulatory ligand has sparked tremendous interest as a target for antibody-directed therapy of solid tumors. This is attributed to high expression of B7-H3 on the surface of tumor and low expression on normal tissues. Natural killer cells are known as key effectors of the innate immune system against cancer and are being explored clinically in a number of immunotherapy settings. Here, we describe a unique platform technology incorporating a cytokine as a bispecific antibody cross-linker to create a tri-specific NK cell engager, or TriKE, directed against the antigen B7-H3. The data shows this immunotherapeutic construct promotes specific natural killer cell expansion. It also induces antibody-dependent cellular cytotoxicity due to simultaneous recognition of both natural killer cells and B7-H3-expressing cancer cells. Finally, using a xenogeneic mouse model, the data shows that the TriKE induces better natural killer cell-mediated control of ovarian cancer. Taken together, the findings presented here indicate that a B7-H3-targeted TriKE has the potential to enhance natural killer cell immunotherapy in solid tumor settings and supports further development.

**Abstract:**

We improved the bispecific antibody platform that primarily engages natural killer (NK) cells to kill cancer cells through antibody-dependent cellular cytotoxicity (ADCC) by adding IL-15 as a crosslinker that expands and self-sustains the effector NK cell population. The overall goal was to target B7-H3, an established marker predominantly expressed on cancer cells and minimally expressed on normal cells, and prove that it could target cancer cells in vitro and inhibit tumor growth in vivo. The tri-specific killer engager (TriKE^TM^) was assembled by DNA shuffling and ligation using DNA encoding a camelid anti-CD16 antibody fragment, a wild-type IL-15 moiety, and an anti-B7-H3 scFv (clone 376.96). The expressed and purified cam1615B7H3 protein was tested for in vitro NK cell activity against a variety of tumors and in vivo against a tagged human MA-148 ovarian cancer cell line grafted in NSG mice. cam1615B7H3 showed specific NK cell expansion, high killing activity across a range of B7-H3+ carcinomas, and the ability to mediate growth inhibition of aggressive ovarian cancer in vivo. cam1615B7H3 TriKE improves NK cell function, expansion, targeted cytotoxicity against various types of B7-H3-positive human cancer cell lines, and delivers an anti-cancer effect in vivo in a solid tumor setting.

## 1. Introduction

Antigen-specific immunotherapies require overexpression of target antigens on tumor cells with minimal off-tumor expression on normal tissues. Ideally, the antigen displays high expression in a broad spectrum of cancers, making the immunotherapy applicable in a number of settings and basket clinical trials are becoming more popular if broad targets can be identified. B7-H3, a transmembrane costimulatory protein that is a member of the B7 family of checkpoint ligands, has gained interest as a target for immunotherapy [1,2,3]. While it is involved both in the context of co-stimulation and inhibition by engaging receptors on T-cells [4], it has also been shown to contribute to immune evasion through expression on antigen presenting cells, such as macrophages, and tumor cells within the tumor microenvironment [5,6,7]. B7-H3 expression is high in many types of cancer but very low in normal tissues [8]. A mouse model, utilizing a B7-H3-targeting CAR T construct that is reactive to mouse cells, has demonstrated anti-tumor responses in the absence of toxicity, further highlighting the safety profile of B7-H3 as a target [9]. Ninety-three percent of ovarian tumors express B7-H3, and expression is associated with advanced stage, high recurrence, and poor survival [10,11]. Similar findings exist for other types of carcinoma including cancer of the colon, prostate, pancreas, non-small-cell lung cancer, and gastric cancer, indicating that B7-H3 may be a useful marker in cancer biology, progression, and therapy across a range of different cancers [12]. Due to these characteristics, there are currently a number of ongoing clinical trials targeting this antigen in modalities ranging from Fc optimized antibodies (NCT02982941) to CAR T cells (NCT04077866).

Bispecific immune engagers such as blinatumomab have shown impressive clinical success [13]. As a single engineered molecule, one of its single chain variable fragments (scFv) targets cancer cells and the other one targets CD3 on T cells. This creates an immune synapse between T cells and cancer cells, resulting in tumor killing. However, activation and proliferation of T cells can result in cytokine release syndrome, disseminated intravascular coagulation, and nervous system events including encephalopathy and seizures [14]. Thus, our group has been interested in selectively engaging natural killer (NK) cells instead of T cells. NK cells are part of the innate immune system, play a major role in tumor surveillance, and have shown potential in a number of studies involving solid tumors and hematologic cancers [15,16,17]. Due to these characteristics, we have designed and described a tri-specific killer engager (TriKE) platform consisting of a single chain variable fragment (scFv) targeting CD16, the most potent activating receptor on NK cells, a scFv targeting a tumor associated antigen, and an IL-15 moiety [18,19,20,21,22]. Recently, we have improved on this platform by adding a single domain antibody against CD16, the result of which is better IL-15 activity and overall function [23]. IL-15 is the most critical homeostatic cytokine for NK cell function [24]. It is necessary for NK cell expansion and survival, it can amplify antibody-dependent cellular cytotoxicity (ADCC), it can induce lymphokine-activated killer activity, and it can enhance production of other co-stimulatory mediators like interferon gamma (IFNγ) and tumor-necrosis factor alpha (TNFα).

In this report, we describe a second-generation TriKE bioengineered with human IL-15 as a modified crosslinker between a humanized camelid anti-CD16 VHH single domain antibody (sdAb) and an anti-B7-H3 scFv, termed cam1615B7H3. Thus, in a single molecule, we were able to merge two important therapeutic properties: the ability to specifically enhance NK cell expansion with the ability to enhance ADCC. cam1615B7H3 demonstrated potent and specific induction of NK cell activity against a variety of solid tumors in vitro while also showing potent activity against in a xenogeneic ovarian cancer model. Thus, targeting B7-H3 with a TriKE may have high therapeutic value in the NK-cell-based immunotherapy of a number of solid cancers.

## 2. Materials and Methods

### 2.1. Construction of cam1615B7H3 TriKEs

Single-domain V_H_H antibodies derived from camelids are known to offer advantages over conventional V_L_-V_H_ scFv fragments. Thus, we spliced the complementary determining regions (CDRs) from a camelid (llama) anti-CD16 [25] into a universal, humanized, heavy chain scaffold [23,26]. This humanized camelid sequence was used to manufacture cam1615B7H3. A hybrid gene encoding cam1615B7H3 was synthesized using DNA shuffling and DNA ligation techniques. The fully assembled gene (from 5′ end to 3′ end) encoded a NcoI restriction site; an ATG start codon; anti-human CD16 VHH; a 20 amino acid (aa) segment, PSGQAGAAASESLFVSNHAY; human wild-type IL-15; the seven amino acid linker, EASGGPE; anti-B7-H3 mAb 376.96 scFv [27]; and a XhoI restriction site. The resulting hybrid gene was spliced into the pET28c expression vector under the control of an isopropyl-D-thiogalactopyranoside (IPTG) inducible T7 promoter. The DNA target gene encoding cam1615B7H3 was 1527 base pairs. The Biomedical Genomics Center, University of Minnesota (St. Paul, MN, USA) verified the gene sequence and the in-frame accuracy of the construct.

### 2.2. Purification of Protein from Inclusion Bodies

*Escherichia coli* strain BL21 (DE3) (Novagen, Madison, WI, USA) was used for the expression of proteins after plasmid transfection. Bacterial expression resulted in the sequestering of target protein into inclusion bodies (IBs). Bacteria were cultured overnight in 800 mL Luria broth containing kanamycin (30 mg/mL). When absorbance reached 0.65 at 600 nm, gene expression was induced with Isopropyl β-D-1-thiogalactopyranoside/IPTG (FischerBiotech, Fair Lawn, NJ, USA). Bacteria were harvested after 2 h. After a homogenization step in a buffer solution (50 mM Tris, 50 mM NaCl, and 5 mM EDTA pH 8.0), the pellet was sonicated and centrifuged. Proteins were extracted from the pellet using a solution of 0.3% sodium deoxycholate, 5% Triton X-100, 10% glycerin, 50 mmol/L Tris, 50 mmol/L NaCl, and 5 mmol/L EDTA (pH 8.0). The extract was washed 3 times.

Bacterial expression in inclusion bodies requires refolding. Thus, proteins were refolded using a sodium N-lauroyl-sarcosine (SLS) air oxidation method (20). IBs were dissolved in 100 mM Tris, 2.5% SLS (Sigma, St. Louis, MO USA) and clarified by centrifugation. Then, 50 μM of CuSO_4_ was added to the solution and then incubated at room temperature with rapid stirring for 20 h for air-oxidization of –SH groups. Removal of SLS was performed by adding 6 M urea and 10% AG 1-X8 resin (200–400 mesh, chloride form) (Bio-Rad Laboratories, Hercules, CA, USA) to the detergent-solubilized protein solution. Guanidine HCl (13.3 M) was added to the solution which was incubated at 37 °C for 2 to 3 h. The solution was diluted 20-fold with refolding buffer, 50 mM Tris, 0.5 M l-arginine, 1 M Urea, 20% glycerol, 5 mM EDTA, pH 8.0. The mixture was refolded at 4 °C for two days and then dialyzed against five volumes of 20 mM Tris-HCl at pH 8.0 for 48 h at 4 °C, then eight volumes for 18 additional hours. The product was then purified over a fast flow Q ion exchange column and further purified by passage over a size exclusion column (Superdex 200, GE, Marlborough, MA, USA). Protein purity was determined with sodium dodecyl sulfate polyacrylamide gel electrophoresis (SDS-PAGE) stained with Simply Blue Safe Stain (Invitrogen, Carlsbad, CA, USA).

### 2.3. Cancer Cell Lines and Antibody

MA-148 (established locally at the University of Minnesota) is a human epithelial high-grade serous ovarian carcinoma cell line. For in vivo experiments, lines were transfected with a luciferase reporter construct using Invitrogen’s Lipofectamine Reagent and selective pressure applied with 10 μg/mL of blasticidin. Ovarian carcinoma cell lines OVCAR5 and OVCAR8 were obtained from the DTP, DCTD Tumor Repository sponsored by the Biological Testing Branch, Developmental Therapeutics Program, National Cancer Institute (NCI), National Institutes of Health (NIH, Frederick, MD, USA). Other cell lines were obtained from the American Type Culture Collection including OVCAR3 (ovarian), C4-2 (prostate), DU145 (prostate), LNCaP (prostate), PC-3 (prostate), A549 (lung), NCI-H322 (lung), NCI-H460 (lung), and Raji cells (Burkitt’s lymphoma). With the exception of Raji cells, used as a negative control, all lines express high levels of B7-H3 (Figure S1A–E). Lines were maintained in RPMI 1640 medium supplemented with 10–20% fetal bovine serum (FBS) and 2 mmol/L L-glutamine. Lines were incubated at a humidified atmosphere containing 5% CO_2_ at a constant 37 °C. When adherent cells were more than 90% confluent, they were passaged using trypsin-EDTA for detachment. For the cell counts a standard hemocytometer was used. Only those cells with a viability >95% were used for the experiments. The sequence for the monoclonal antibody scFv fragment 376.96 was obtained by Dr. Ferrone and used to construct the TriKE.

### 2.4. Cell Products

Peripheral blood mononuclear cells (PBMCs) were obtained from normal volunteers or patients after consent was received, and institutional review board (IRB) approval was granted (protocols 9709M00134 and 1607M91103), in compliance with guidelines by the Committee on the Use of Human Subjects in Research and in accordance with the Declaration of Helsinki. For in vivo studies, fresh PBMCs were magnetically depleted three times (i.e., three passthroughs across the magnet) of CD3 and CD19-positive cells, according to the manufacturer’s recommendations (STEMCELL Technologies, Cambridge, MA, USA), to generate an NK-cell-enriched product. Ovarian cancer specimens (ascites) were collected in women diagnosed with advanced-stage ovarian or primary peritoneal carcinoma at time of primary debulking surgery. For prostate cancer, blood was obtained from two patients with metastatic castration resistant prostate cancer and one patient with metastatic hormone sensitive prostate cancer. For lung cancer, blood was obtained from seven unresectable lung cancer patients at the time of diagnosis, prior treatment. Cells were pelleted, lysed for red blood cells, cryopreserved in 10% DMSO/90% FBS, and stored in liquid nitrogen.

### 2.5. NK Cell Expansion via IL-15 Stimulation

To measure the ability of the TriKE to specifically induce NK cell expansion via the IL-15 moiety, PBMCs from healthy donors were labeled with CellTrace Violet Proliferation Dye (Invitrogen, Carlsbad, CA, USA) according to kit specifications. After staining, cells were cultured with TriKEs at noted concentrations, or equimolar concentrations of controls, and incubated in a humidified atmosphere containing 5% CO_2_ at 37 °C for seven days. Cells were harvested, stained for viability with Live/Dead reagent (Invitrogen, Carlsbad, CA, USA) and surface stained for anti-CD56 PE/Cy7 (Biolegend, San Diego, CA, USA) and anti-CD3 PE-CF594 (BD Biosciences, Franklin Lakes, NJ, USA) to gate on the viable CD56^+^ CD3^-^ NK cell population or the CD56^-^CD3^+^ T cell population. Data analysis was performed using FlowJo software (FlowJo LCC, version 7.6.5, Ashland, OR, USA).

### 2.6. Evaluation of Cytotoxicity and NK Cell Activation

ADCC was measured in a flow cytometry assay by evaluating degranulation via CD107a (lysosomal-associated membrane protein LAMP-1) and intracellular IFNγ production. Upon thawing, normal donor and patient-derived PBMCs or ascites cells were rested overnight (37 °C, 5% CO_2_) in RPMI 1640 media supplemented with 10% fetal calf serum (RPMI-10). The next morning, they were suspended with tumor-target cells or media after washing twice with RPMI-10. Cells were then incubated with TriKEs or controls for 10 min at 37 °C. Fluorescein isothiocyate (FITC)-conjugated anti-human CD107a monoclonal antibody (BD Biosciences, San Jose, CA, USA) was then added. Following an hour 37 °C incubation, GolgiStop (1:1500, BD Biosciences) and GolgiPlug (1:1000, BD Biosciences) were added for 3 h. After washing with phosphate buffered saline, the cells were stained with PE/Cy 7–conjugated anti-CD56 mAb, APC/Cy 7–conjugated anti-CD16 mAb, and PE-CF594–conjugated anti-CD3 mAb (BioLegend, San Diego, CA, USA). Cells were incubated for 15 min at 4 °C, washed, and fixed with 2% para-formaldehyde. Cells were then permeabilized using an intracellular perm buffer (BioLegend) to evaluate production of IFNγ through detection via aBV650 conjugated anti-human IFNγ antibody (BioLegend). Samples were washed and evaluated in an LSRII flow cytometer (BD Biosciences, San Jose, CA, USA).

### 2.7. Real-Time Tumor-Killing Assay

Tumor killing was evaluated in real-time using the IncuCyte platform. Magnetic-bead-enriched CD3-CD56+ NK effector cells were plated into 96-well flat clear-bottom polystyrene tissue-culture-treated microplates (Corning, Flintshire, UK) along with NuclightRed stably expressing OVCAR8 cells at a 2:1 effector:target ratio. Caspase-3/7 green dye (Sartorious, Ann Arbor, MI, USA) was added to pick up dying cells that have not yet lost NuclightRed fluorescence. Noted treatments were then added at a 30 nM concentration, and the plate was placed in an IncuCyte ZOOM^®^ platform housed inside a cell incubator at 37 °C/5% CO_2_. Images from three technical replicates were taken every 15 min for 48 h using a 4X objective lens and then analyzed using IncuCyte™ Basic Software v2018A (Sartorious). Graphed readouts represent percentage live OVCAR8 targets (NuclightRed^+^Caspase-3/7^−^), normalized to live targets alone at the starting (0 h) time point.

### 2.8. Mass Cytometry (CyTOF)

For mass cytometry (CyTOF) studies, PBMCs were incubated alone or with OVCAR8s at a 2:1 ratio +/− cam1615B7H3 (30 nM) for 24 h. After harvesting samples, cells were counted, and viability was measured using trypan blue exclusion. Two hundred thousand cells from each donor were aliquoted into 5-mL polystyrene U-bottom tubes for barcoding and CyTOF staining. Cells were stained with Cisplatin (Fluidigm Product# 201064, San Francisco, CA, USA), followed by barcoding using the Cell-ID 20-Plex Pd Barcoding Kit (Fluidigm Product# 201060). After barcoding, all cells were combined into a single 5-mL polystyrene U- bottom tube and incubated in the surface marker antibody cocktail for 30 min at 4 °C.

Following surface staining, cells were then fixed using 2% PFA. For intracellular staining, cells were permeabilized by incubation with Triton X 0.1% for 5 min at room temperature, followed by incubation with intracellular antibody cocktail for 30 min at 4 °C. Stained cells were then incubated overnight with Cell-ID Intercalator (Fluidigm Product# 201192A). The following morning cells were washed and run on the CyTOF 2 instrument. Wash steps were completed using either Maxpar PBS (Fluidigm Product# 201058), Maxpar Cell Staining Buffer (Fluidigm Product# 201068), or Millipure Water at 1600RPM for 4 min. For custom tagged antibodies: Conjugation of heavy metals to a specific ScFv is conducted using the Maxpar antibody labeling kit (Fluidigm). The protocol involves partial antibody reduction using 0.5 M TCEP: Pierce Bond-Breaker TCEP Solution (Thermo Scientific Product# 77720, Waltham, MA, USA), as well as comprehensive buffer exchange using centrifugal filter units of both 3kDa and 50kDa size (Millipore Product# UFC500396, UFC505096, Burlington, MA, USA). After conjugation of the antibody, yield is measured, and the final reagent is stored in antibody stabilizer (Boca Scientific Product# 131 000, Westwood, MA, USA). The reagent is then titrated and verified against known flow cytometry antibodies. Data from the three donors was concatenated. FCS file concatenation was completed with a combination of Cytobank and Flowjo. All Visne analyses were carried out in Cytobank.

### 2.9. In Vivo Mouse Study and Imaging

MA-148-Luc ovarian cancer cells were incorporated into a previously described NK cell xenogeneic mouse model system [28]. NSG mice (NOD.Cg-Prkdc^scid^ Il2rg^tm1Wjl^/SzJ, n = 5/group) were injected IP with 2.0 × 10^5^ MA-148-luc cells and then three days later conditioned with low-dose total body irradiation (225 cGy). The following day, all groups received highly enriched NK cells (PBMC magnetically CD3 and CD19 depleted), equivalent to 1 million NK cells/mouse, and were started on the drug regimen. A single course of treatment consisted of an IP injection of 30 μg of TriKE or 5 µg rhIL-15 given every day of the week (Monday–Friday) for three weeks. MA-148-luc cells are a subline of MA-148 that have been transfected with a luciferase reporter gene, allowing for imaging of the mice each week to determine their bioluminescent activity and to monitor tumor progression. Briefly, mice were injected with 100 μL of 30 mg/mL luciferin substrate 10 min prior to imaging and then anesthetized via inhalation of isoflurane gas (25). Mice were then imaged using the Xenogen Ivis 100 imaging system and analyzed with Living Image 2.5 software (Xenogen Corporation, Alameda, CA, USA). At the end of the experiment (day 21), all the animals were sacrificed, and postmortem peritoneal lavages were performed to analyze human NK cell content by flow cytometry. Animal imaging and analysis was performed at the University of Minnesota Imaging Center. Mouse studies were carried after approval (protocol 1908-37330A) from the Institutional Animal Care and Use Committee (IACUC) at the University of Minnesota and in compliance with their guidelines.

### 2.10. Statistical Analysis

GraphPad PRISM 8 (GraphPad Prism Software, Inc., San Diego, CA, USA) was used to create all statistical tests. For all in vitro studies, one-way ANOVA with repeated measures was used to calculate significance in comparisons to the cam1615B7H3 group. For mouse studies, two-way ANOVA was used to calculate significance in the longitudinal study, while one-way ANOVA was used to calculate the significance in differences in radiance at the day-21 timepoint. An unpaired *t* test was used to evaluate differences in cell counts and MFI. Bars represent mean ± SEM. Statistical significance is displayed as * *p* < 0.05, ** *p* < 0.01, *** *p* < 0.001, and **** *p* < 0.0001.

## 3. Results

### 3.1. Creation and Purification of a B7-H3 Targeting TriKE

**Figure 1:**
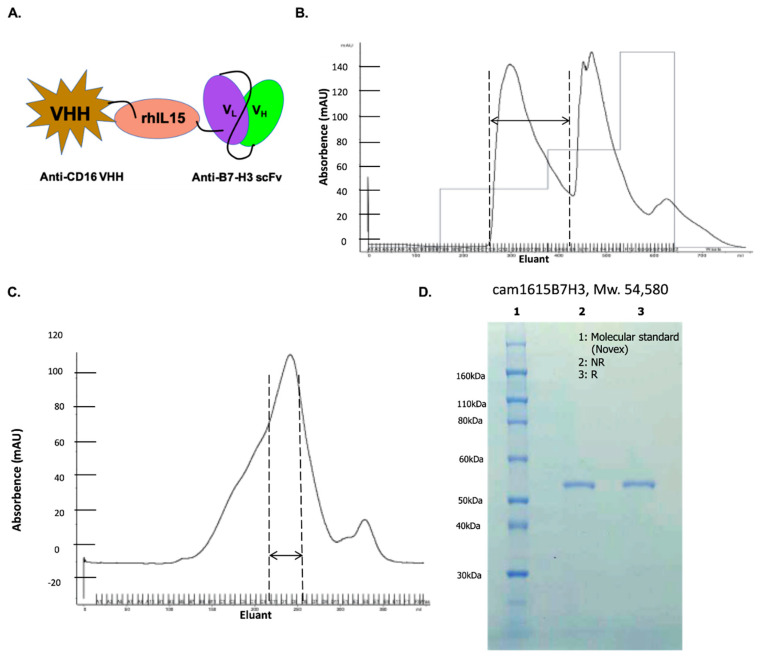
Construction and isolation of cam1615B7H3 tri-specific killer engager (TriKE). (**A**) Schema of the TriKE construct consisting of (left to right) camelid anti-CD16 VHH, Human IL-15, and anti-B7-H3 scFv. (**B**) Chromatography trace from the first-step purification of cam1615B7H3 on an ion exchange (FFQ) column. The collection peak is indicated by the double-sided arrow. (**C**) Chromatography trace from the second-step purification of cam1615B7H3 on a size exclusion chromatography (SEC) column. The collection peak is indicated by the double-sided arrow. (**D**) sodium dodecyl sulfate polyacrylamide gel electrophoresis (SDS-PAGE) gel indicating the purity of the final product after the two orthogonal column steps. The lanes of the gel display molecular marker, non-reduced (NR) product, and reduced (R) product.

In order to construct a second-generation TriKE capable of both ADCC and NK cell expansion, we modified our previously reported TriKE platform [22]. A wild-type human IL-15 crosslinker with two modified flanking regions was inserted between two antibody fragments—an n-terminal VHH humanized camelid anti-CD16 fragment [23] and a c-terminal anti-B7-H3 fragment [27]—creating cam1615B7H3. Figure 1A shows a schematic of the B7-H3 TriKE construct. Figure 1B shows the absorbance tracing from the FFQ ion exchange column as the first phase of the purification with the eluant collected in 8-mL aliquots shown on the abscissa of the graph. The double-sided arrow shows the collection peak as drug exits the column. Figure 1C shows the absorbance tracing from the second purification phase, size exclusion chromatography (SEC). The first peak off the column was collected and the various drug containing fractions were pooled and analyzed using SDS-PAGE with Comassie Blue staining for the presence of a uniform product (Figure 1D). The final product was greater than 90% pure with a molecular weight of about 55 kDa; the predicted molecular weight was 54.58 kDA. As with other TriKE molecules, this TriKE is expected to have a rapid clearance profile due to its size, with EC50 ranges in the order of a couple of hours [23].

### 3.2. cam1615B7H3 TriKE Induces Potent and Specific NK Cell Proliferation

**Figure 2:**
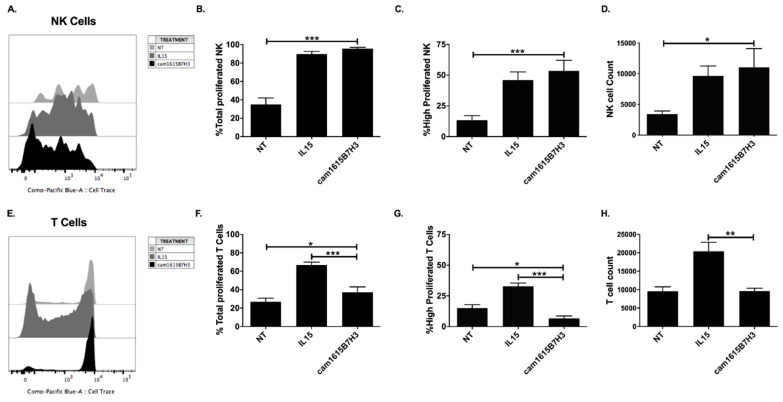
cam1615B7H3 TriKE induces potent, and specific, natural killer (NK) cell proliferation. Peripheral blood mononuclear cells (PBMCs) were CellTrace Violet labeled and incubated for seven days at 5 nM equimolar concentrations of rhIL-15 or cam1615B7H3 TriKE. On day 7, the cells were harvested and stained for flow cytometric evaluation. (**A**) A representative histogram showing NK cell (CD56^+^ CD3^−^) proliferation measured as the dilution of the CellTrace Violet dye. Pooled data showing (**B**) the overall proportion of NK cells that proliferated or (**C**) the proportion of NK cells that highly proliferated (measured as proliferation beyond three divisions), as well as (**D**) NK cell counts in the cultures (N = 9). In comparison, (**E**) a representative histogram showing T cell (CD56^−^ CD3^+^) proliferation. (**F**) Pooled data showing the overall proportion of T cells proliferated or (**G**) the proportion of T cells highly proliferated, as well as (**H**) T cell counts in the cultures (N = 9). * *p* < 0.05, ** *p* < 0.01, *** *p* < 0.001.

The wild-type IL-15 moiety in the cam1615B7H3 TriKE is designed to induce targeted delivery of a proliferative signal to NK cells. To test this, we carried out proliferation assays evaluating dilution of a CellTrace dye over a 7-day period on PBMCs treated with no treatment (NT), monomeric rhIL-15 (IL15), or the TriKE (cam1615B7H3). At the end of the seven days, cells were harvested and proliferation was evaluated by gating on CD56^+^CD3^−^ cells. While no treatment (NT) resulted in low proliferation with low NK cell numbers, the cam1615B7H3 induced an overall increase in proliferation that was similar in amplitude to that induced by rhIL-15 (Figure 2A–D), with no significant differences between those two groups. Since IL-15 acts on both NK cells and T cells, specificity was evaluated next by gating on T cells (CD56^−^CD3^+^). Minimal T cell proliferation was seen in the TriKE treatment group in contrast to rhIL-15, which induced robust proliferation of T cells (Figure 2E,F) clearly showing that the cam1615B7H3 TriKE IL-15 delivery was more restricted to NK cells. This differential was particularly notable in robustly proliferating populations (past three divisions), where the cam1615B7H3 TriKE showed significantly less proliferation than the untreated group (Figure 2G), while T cell numbers did not differ between the untreated and TriKE-treated groups (Figure 2H). This data indicates that the cam16 engager in the cam1615B7H3 TriKE is specifically delivering IL-15 to NK cells and not T cells.

### 3.3. cam1615B7H3 TriKE Exhibits Potent Killing of Ovarian Cancer

**Figure 3:**
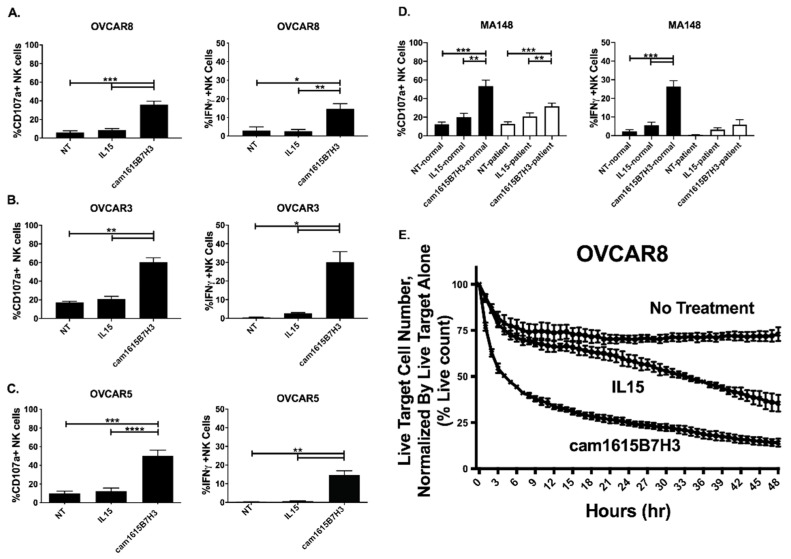
cam1615B7H3 TriKE enhances NK cell function against ovarian tumor targets. Healthy donor or ovarian cancer PBMC cells were incubated with ovarian tumor targets at 2:1 E:T ratio for 4hrs with 30 nM TriKE or control condition. Pooled proportion of NK cells from healthy donors expressing CD107a+ (degranulation) or IFNγ cytokine production against (**A**) OVCAR8 targets (N = 8), (**B**) OVCAR3 targets (N = 4), and (**C**) OVCAR5 targets (N = 7). (**D**) Pooled analysis of proportion of NK cells from healthy donors (black bars) or ovarian cancer patients (white bars) expressing CD107a+ or IFNγ against MA-148 targets (N = 9 and N = 6 respectively). (**E**) Tumor killing was evaluated using IncuCyte imaging assay. NuclightRed expressing OVCAR8 targets were incubated with enriched healthy donor NK over 48hrs, with Caspase 3/7 viability dye. The percentage of Live (Nuclight Red+Caspase 3/7−) tumor cells was quantified over a 48-h period and normalized to tumor alone. Readings were obtained every 15 min (representative of four separate experiments). * *p* < 0.05, ** *p* < 0.01, *** *p* < 0.001, and **** *p* < 0.0001.

The ability of cam1615B7H3 TriKE to mediate NK cell activity against ovarian cancer cells was evaluated next. Ovarian cancer cells used displayed robust B7-H3 expression (Figure S1B). Since B7-H3 has been shown to have a role in immune responses, the capacity of the cam1615B7H3 to induce activity against normal immune cells was evaluated in PBMCs. Flow cytometric assays, allowing for gating on NK cells, determined that the cam1615B7H3 induced some background degranulation (CD107a) on NK cells in comparison to controls, but this activity was low (Figure S2A). No background noise was seen with IFNγ. In contrast, when PBMCs were incubated with a variety of high grade serous ovarian adenocarcinoma cell lines, including OVCAR8, OVCAR3, and OVCAR5, robust NK cell degranulation and intracellular IFNγ production was seen compared to no treatment and rhIL-15 alone (Figure 3A–C). In order to determine if the individual components of the TriKE could induce NK cell activity on their own, the individual cam16 VHH, IL-15, or anti-B7-H3 scFv components were incubated with PBMCs and OVCAR8 cells and activity was determined (Figure S2B). The data clearly shows that individual components do not enhance NK cell activity against OVCAR8 cells. NK cell activity, from normal donor PBMCs and ascites from the peritoneal cavity of ovarian cancer patients at the time of surgery, was assessed against MA-148 cells, another high-grade serous ovarian adenocarcinoma cell line (Figure 3D). Compared to controls, the cam1615B7H3 TriKE induced robust activity on normal donor NK cells. While NK cell activity from ovarian-cancer-derived ascites samples was decreased, as expected due to alterations in NK cell function driven by the tumor microenvironment and decreases in CD16 expression shown in our previous studies [28], the cam1615B7H3 TriKE induced significantly enhanced NK cell degranulation compared to controls. Finally, killing of ovarian cancer tumor cells (OVCAR8s) was measured dynamically over a two-day period in the presence of enriched NK cells alone (No Treatment), NK cells and rhIL-15 (IL15), and NK cells and the cam1615B7H3 TriKE (Figure 3E). In this assay, tumor cells can be tracked with a stably expressed fluorescent protein (NucLight Red) and detection of early apoptosis, used to exclude recent cell death, is mediated by a green fluorescent Caspase3/7 dye. The basic readout provided is the number of tumor cells alive (Red^+^Green^−^) normalized to tumor alone at the noted times. As shown, the cam1615B7H3 TriKE induced robust and rapid tumor killing when compared to controls. This data indicates that the cam1615B7H3 TriKE potently enhances activity against ovarian cancer cells in vitro. Of note, the cam1615B7H3 TriKE induced similar degranulation and stronger IFNγ production against ovarian cancer when compared to a potent natural cytotoxicity signal, in the absence of TriKE, induced by K562 cells (Figure S2E). Fold NK cell activation against all ovarian cancer cell lines, calculated as activation on PBMC+Tumor+TriKE divided by activation on PBMC+TriKE alone, was higher than activation by the B7-H3-negative Raji line (Figure S2F), indicating the B7-H3 specificity of the TriKE.

### 3.4. cam1615B7H3 TriKE Targets Prostate Cancer

**Figure 4:**
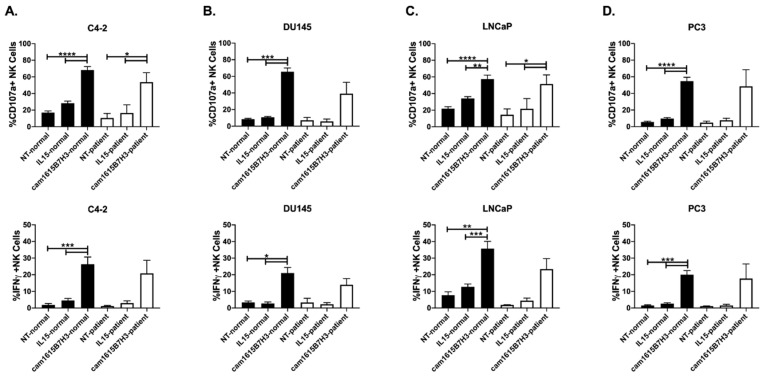
cam1615B7H3 TriKE enhances NK cell function of healthy donors (black bars) and prostate cancer patients (white bars) against prostate tumor targets. Pooled analysis of proportion of NK cells from healthy donors or prostate cancer patients expressing CD107a+ (top) or IFNγ (bottom) against (**A**) C4-2, (**B**) DU145, (**C**) LNCaP, and (**D**) PC3 targets (N = 9 for healthy donors, N = 3 for patients). * *p* < 0.05, ** *p* < 0.01, *** *p* < 0.001, and **** *p* < 0.0001.

The ability of the cam1615B7H3 TriKE to improve NK cell activity against prostate cancer was tested next. As with ovarian cancer cells, all of the prostate cancer cell lines tested expressed B7-H3 (Figure S1C). For these tests, we utilized normal donor PBMCs and PBMCs obtained from metastatic prostate cancer patients. While metastatic prostate cancer patients displayed a slight decrease in NK cell activity, when compared to normal donors, the cam1615B7H3 TriKE enhanced degranulation and IFNγ production, in both normal donor and patient NK cells, against C4-2, DU145, LNCaP and PC3 prostate cancer adenocarcinoma cell lines when compared to the controls (Figure 4A–D). As with ovarian cancer, the individual cam16 VHH or anti-B7-H3 scFv components did not induce increased NK cell activation against C4-2s (Figure S2C). Thus, the data indicates that the cam1615B7H3 TriKE has promise in NK cell immunotherapy within the prostate cancer setting and shows that the NK cell function can be rescued on patients who require novel interventions due to poor outcomes with current therapeutic approaches. The signal induced by the TriKE with prostate cancer cells was stronger than that induced by a strong natural cytotoxicity signal and was specific to B7-H3 (Figure S2E,F).

### 3.5. cam1615B7H3 TriKE Targets Lung Cancer

**Figure 5:**
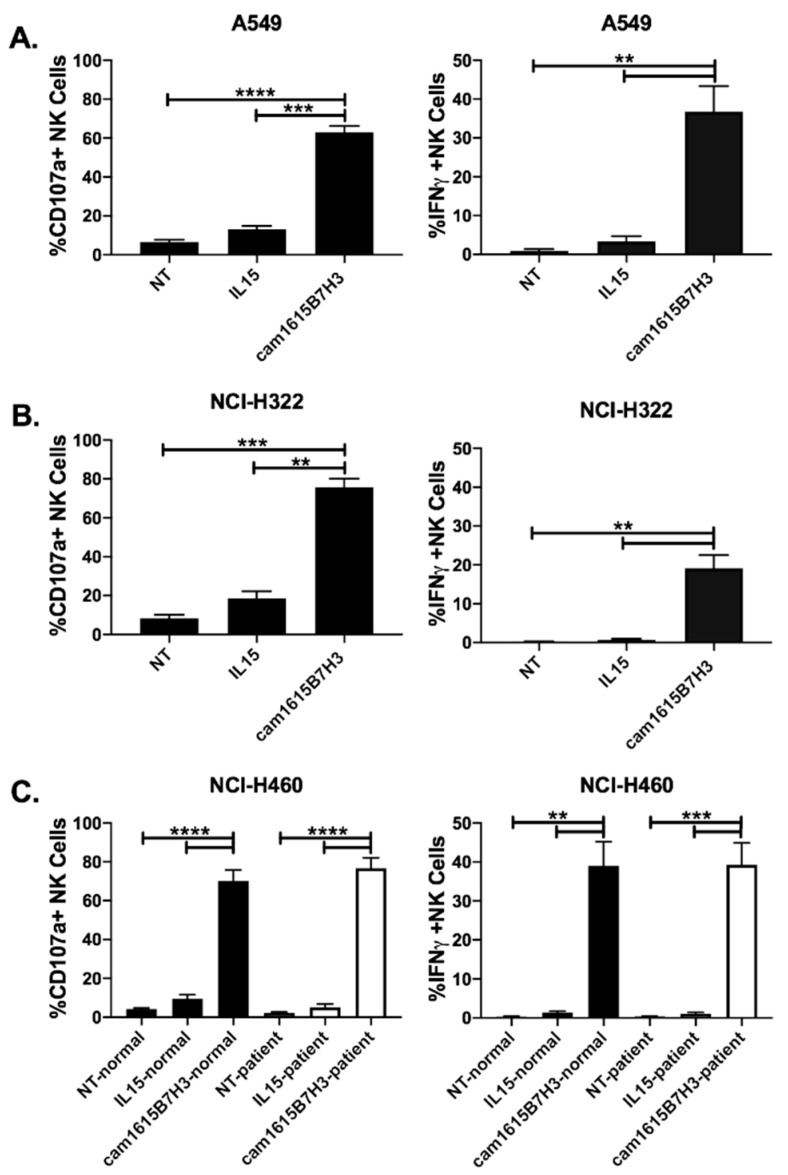
cam1615B7H3 TriKE enhances NK cell function against lung tumor targets. Pooled analysis of proportion of NK cells from healthy donors expressing CD107a+ (**left**) or IFNγ (**right**) against (**A**) A549 (N = 6) and (**B**) NCI-H322 (N = 5) tumor targets. (**C**) Pooled proportion of NK cells from healthy donors (black bars) or lung cancer patients (white bars) expressing CD107a+ or IFNγ against NCI-H460 (N = 7). ** *p* < 0.01, *** *p* < 0.001, and **** *p* < 0.0001.

The ability of cam1615B7H3 TriKE to improve NK cell activity against B7-H3-expressing lung cancer (Figure S1D) was tested on normal donor PBMCs incubated with A549 and NCI-H322, two non-small cell lung cancer adenocarcinoma lines (Figure 5A,B). In both instances the cam1615B7H3 TriKE significantly and robustly improved NK cell activation when compared to controls. Individual cam16 VHH or anti-B7-H3 scFv components were tested and showed no background NK cell activity against A549s (Figure S2D). Normal donor PBMCs as well as PBMCs from patients with newly diagnosed unresectable lung cancer, prior to any therapy, were incubated with NCI-H460 cells, a large cell lung cancer cell line. As the data clearly shows, cam1615B7H3 treatment strongly increased NK cell function, on both normal donor and patient samples, against large cell lung cancer when compared to controls (Figure 5C). The TriKE-mediated induction of NK cell degranulation and IFNγ production against lung cancer cells was higher than that seen when NK cells are incubated with K562 targets alone (Figure S2E). Activation against lung cancer cell lines was specific to B7-H3 expression as it was higher than activation by B7-H3^−^ Raji cells (Figure S2E). Thus, the data indicates that the cam1615B7H3 TriKE has broad B7-H3-specific activity against a number of solid tumor targets.

### 3.6. High Dimensional Analysis of cam1615B7H3 TriKE Activated Cells

**Figure 6:**
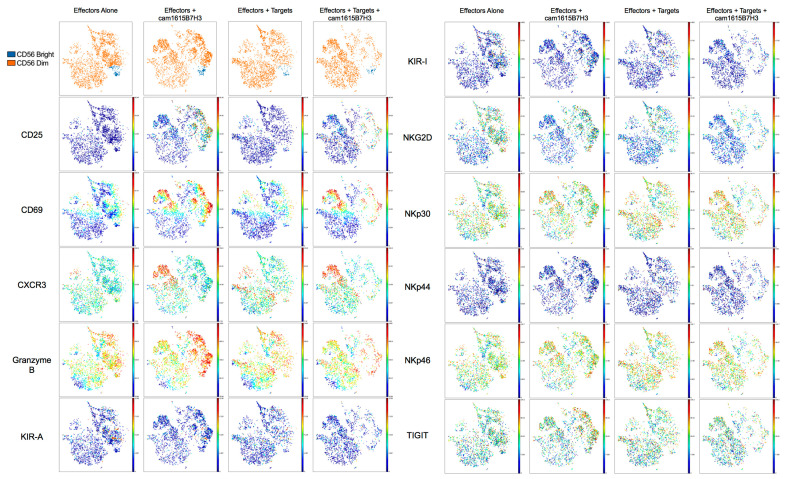
High dimensional analysis of cam1615B7H3 activated NK cells. Concatenated analysis of three donors PBMCs incubated alone, with 30 nM cam1615B7H3 TriKE, with OVCAR8 tumor (2:1 E:T), or with 30 nM cam1615B7H3 TriKE and OVCAR8 tumor. Data was visualized in viSNE (Cytobank) and gated on CD45^+^CD56^+^CD3^−^ cells. With the exception of the CD56 plot, color represents protein intensity (red = highest, and dark blue = lowest).

To broadly evaluate the phenotypic and functional effects of TriKE activation on NK cells, we utilized a custom, 42 parameter, CyTOF (mass cytometry) NK cell targeted panel. PBMCs were left untreated, incubated with cam1615B7H3 TriKE for 24 h, incubated with tumor (OVCAR8s) for 24 h, or incubated with tumor and cam1615B7H3 TriKE for 24 h. Cells were then stained, fixed, and run on a CyTOF2. Samples (three biologic replicates per condition) were concatenated, and data was visualized with viSNE, which uses all expression information to display localization of individual cells in a 2D plot in order to explore the multidimensional data (Figure 6). Our data indicated no changes in distributions of CD56^brights^ versus CD56^dims^. Activation markers CD25 and CD69 were both induced with TriKE treatment, as was the chemokine receptor CXCR3. Granzyme B, involved in cytolytic activity of NK cells, was primed on effectors + TriKE, but in the presence of tumor targets (effectors+tumor+TriKE) these Granzyme B high cells disappeared, likely as a cause of ADCC driven specific degranulation. Interestingly, both inhibitory KIR (KIR2DL1, KIR2DL3 and KIR3DL1) and activating KIR (KIR2DS1 and KIR2DS4) were reduced in expression when effectors were exposed to tumor in the presence of TriKE. This also seemed to be the case with NKG2D, but the natural cytotoxicity receptors (NCR: NKp30, NKp44, and NKp46) were less affected. Finally, the inhibitory receptor TIGIT also did not seem as affected. Taken together, this data demonstrates dynamic changes in NK cell phenotype post TriKE mediated activation.

### 3.7. cam1615B7H3 TriKE Mediates Anti-Tumor Activity In Vivo

Determination of in vivo activity is a critical step for translation. However, prior to evaluating the ability of the cam1615B7H3 TriKE to induce function against tumor the potential for toxicity was assessed. To do this NSG mice were irradiated, engrafted with 1 million NK cells, treated with nothing, IL-15, or cam1615B7H3 for three weeks, and weights were tracked over the course of 90 days post initial treatment (Figure S3A). Despite an initial drop in weight in all groups, likely due to the irradiation, no significant differences were seen in the TriKE treated group vs. the controls. This is perhaps not surprising given the low toxicity profile of IL-15 and the safety profile of B7-H3 [9,29].

**Figure 7:**
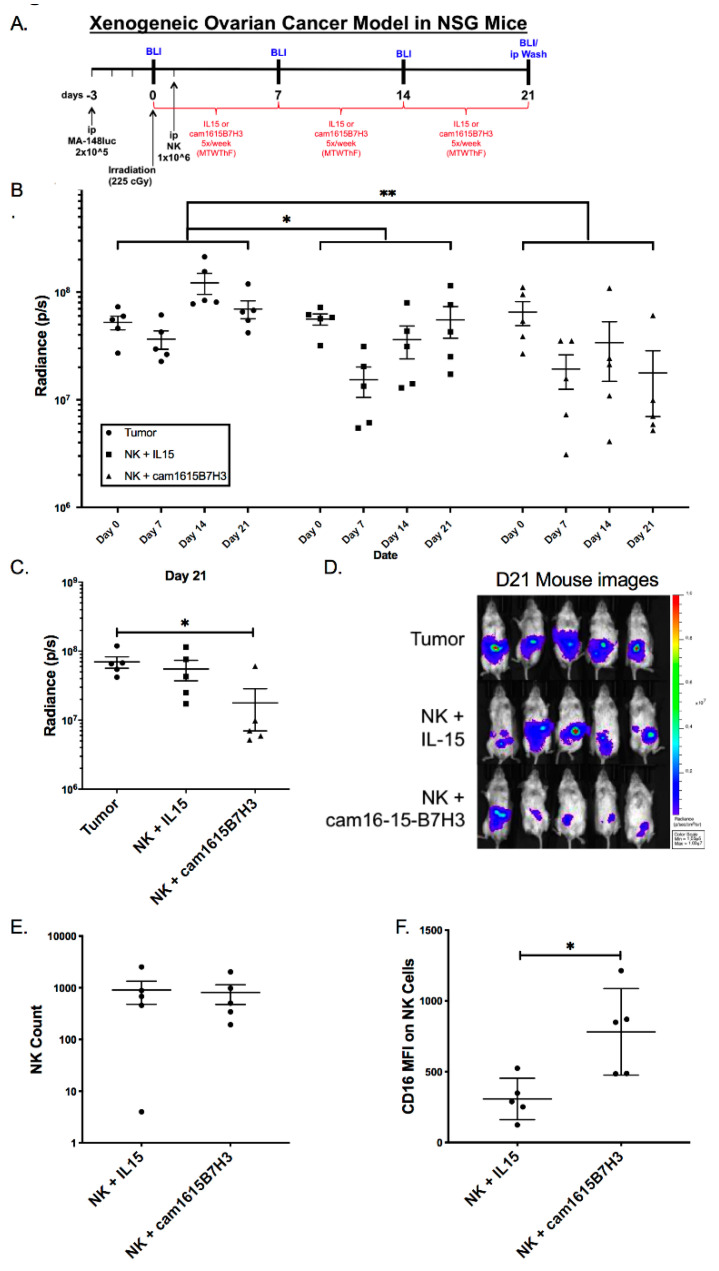
cam1615B7H3 TriKE is efficacious in subduing ovarian tumor progression in vivo. (**A**) Diagram showing Xenogeneic ovarian cancer MA-148-Luc model in female NSG mice (N = 5 per treatment group). (**B**) Bioluminescent imaging indicative of tumor progression, measured as Total flux radiance (p/s), over three weeks in the MA148 mouse model treated with enriched NK cells and noted treatments. (**C**) Bioluminescent imaging results on day 21 data point. (**D**) Graphed total flux radiance (p/s) on day 21. (**E**) Scatter plot of CD56+CD3− NK cell numbers from peritoneal lavages for the rhIL-15 and cam1615B7H3 TriKE treatment groups at D21. (**F**) Scatter plot of CD16 media fluorescence intensity on NK cells from peritoneal lavages for the rhIL-15 and cam1615B7H3 TriKE treatment groups at day 21. * *p* < 0.05, ** *p* < 0.01.

The in vitro data indicates that the cam1615B7H3 TriKE can potently activate NK cells against a variety of tumors, but to evaluate whether this TriKE has efficacy in a pre-clinical model, we used a previously described xenogeneic mouse model of ovarian cancer (Figure 7A). In this model, human NK cells and human high grade serous MA-148-luc cells are injected into the peritoneal cavity of NSG mice [28,30]. Longitudinal analysis of tumor progression showed the cam1615B7H3 treated mice displayed the lowest tumor progression, when compared to the IL-15 treated or tumor only mice (Figure 7B). At the time of harvest (day 21), the cam1615B7H3-treated mice had significantly lower tumor burden than the tumor only group (Figure 7C,D). Peritoneal lavages at this timepoint showed similar human NK cell numbers in the rhIL-15 and cam1615B7H3 treated groups indicating that the differences in tumor control were not driven by differences in NK cell numbers alone (Figure 7E). Relevant to the mechanism of action of the cam1615B7H3 TriKE, TriKE-treated mice had NK cells with higher levels of CD16 expression than IL-15 treated mice (Figure 7F). PD-1 expression, often associated with exhaustion in immune cells, also had a lower (but not significant) trend of expression in the TriKE-treated vs. the IL-15-treated mice (Figure S3B).

## 4. Discussion

Ideal targeted immunotherapeutic interventions for solid tumors will have broad-spectrum recognition of a variety of carcinomas with limited or no on-target off-tumor toxicities. B7-H3 displays these characteristics: it has high expression in a number of tumors and low expression in normal tissues [31]. Targeted antibody-based therapies for B7-H3 are currently being explored in the clinic (NCT04185038, NCT02982941, NCT03406949, NCT03729596, NCT04077866, and NCT02475213). Both the safety profile and efficacy of anti-B7-H3 antibodies in clinical trials thus far have been favorable. Radiolabeled antibodies targeting B7-H3 have been safely administered for at least 10 years [32,33]. The drug has been deemed safe enough to use intracranially in children [34]. Interestingly, B7-H3 reportedly is expressed on vasculature and stroma fibroblasts, indicating that this antigen could be used to target the tumor vasculature and architecture [35]. A clear correlation exists between high B7-H3 expression and various tumor growth parameters, including fewer tumor-infiltrating lymphocytes, faster cancer progression, and poor clinical outcome in several cancers such as pancreatic ductal adenocarcinoma (PDAC), prostate cancer, ovarian cancer, lung cancer, and clear cell renal carcinoma [8,11,36,37,38,39]. Furthermore, natural cytotoxicity against most cancers is usually not enough for endogenous NK cells to keep cancer progression at bay, highlighted by low natural cytotoxicity against most tumor lines tested in this study. Taken together, these studies make a very compelling case for targeting B7-H3.

None of the previous therapeutic approaches, however, combine cytokine signaling and ADCC, two critical components for optimal NK cell immunotherapy. The cam1615B7H3 protein described here uses that optimal combination. Our data indicates that the cam1615B7H3 TriKE delivers a specific IL-15 signal to the NK cells, preventing off target toxicities, and also mediates ADCC against a variety of adenocarcinoma cell lines in the ovarian, prostate, and lung cancer settings [40,41,42,43]. This dual mechanism of action allows for enhanced NK cell proliferation, survival, and targeted activation. Our previous studies, comparing TriKEs to bi-specific killer engagers (BiKEs) lacking IL-15, have shown that the IL-15 moiety in the TriKE induces NK cell proliferation, survival, increased STAT5 signaling, and enhanced priming [22,44]. We should note, however, that our in vitro studies show some induction of overall T cell proliferation by the TriKE, albeit minimal in nature when compared to treatment with an equimolar concentration of IL-15. This indicates that, while TriKE is inducing more specificity than monomeric IL-15, it still triggers T cell proliferation at a low level. Interestingly, while overall T cell proliferation when compared to no treatment is increased by the TriKE, proliferation beyond three divisions is actually decreased, and there are no differences in T cell numbers at the end of culture when comparing these two groups. Exploration in more complex models and patients will be needed to fully outline the specificity of the cam1615B7H3 TriKE and evaluate the impact on T cells and, more importantly, T cell toxicities.

While pre-clinical ovarian cancer mouse model results are encouraging and treated animals had stable disease, the treatment was not curative within this model. This may be due to various factors. Human NK cell donors are variable, a problem that may be solved by breakthroughs in NK cellular products like induced pluripotent stem cell derived NK cells (iNK). Also, the TriKE molecule is small, less than 65 kDa in size, resulting in quick clearance through the kidney and sub-optimal dosing. Different donors might clear at different rates. Alternatively, NK cell exhaustion, either mediated by IL-15 or through strong NK cell activation, could be operant [45,46]. TriKEs are dependent on targeting CD16 for activation and can be cleaved by the metalloproteinase ADAM17 [47]. We and others have previously described low levels of CD16 in the NK cells derived from the ascites of women with ovarian cancer and the MA-148 xenogeneic mouse model mimics this phenomenon [28,48]. CD16 cleavage might be mediated by either over-activation of the NK cells by the tumor itself or the inflammatory tumor microenvironment, as ADAM17 can be triggered by both activating and cytokine receptors. This is not unique to ovarian cancer, as reduced CD16 expression on NK cells has been described in other tumor settings [49,50]. Though the CD16 downmodulation may not be seen in every tumor setting, our ascites data indicates that in settings with low CD16 expression the TriKEs can still mediate tumor killing, albeit in a reduced fashion. However, combination with ADAM17 inhibitors, which have been clinically tested for years, or cellular products that have uncleavable CD16 receptors, recently described and currently being clinically tested (NCT04023071), should greatly improve the activity of TriKEs in settings where CD16 is down-regulated [51,52,53].

## 5. Conclusions

While the majority of immunotherapy modalities focus on checkpoint blockade and T cells, natural killer cells have a number of characteristics that make them ideal candidates for cell-based therapy against solid tumors. This study focuses on a unique biologic platform technology, incorporating IL-15 as a bispecific antibody cross-linker, to drive NK-cell-mediated targeting of a broad spectrum of cancers. TriKEs overcome non-specific mechanisms of natural cytotoxicity by promoting an antigen-specific synapse intended to enhance functional NK cell-mediated killing, activation, and proliferation. The TriKE molecule described in this study targets B7-H3, a member of the B7 costimulatory family of Ig proteins that is overexpressed in a number of solid tumor malignancies. We find that B7-H3 is a robust target for TriKE molecules, selectively boosting NK-cell in vitro killing of ovarian cancer, prostate cancer, and lung cancer. The IL-15 action is remarkably specific to NK-cell activity with little off-target effects on T cells. Of translational relevance, this manuscript provides the first in vivo xenograft data, supporting the notion that TriKEs can work against solid tumors and supports their future clinical development.

## Supplementary Materials

The following are available online at https://www.mdpi.com/2072-6694/12/9/2659/s1, Figure S1: B7-H3 expression on cancer cell lines. Figure S2: (A) background induction of NK cell activation by TriKE; (B-D) induction of NK cell activation by individual components of the TriKE; (E-F) Comparison of TriKE mediated activation against different cell lines. Figure S3: TriKE mediated in vivo toxicity and exhaustion.

## References

[1] Greaves P., Gribben J.G. (2013). The role of B7 family molecules in hematologic malignancy. Blood.

[2] Chapoval A.I., Ni J., Lau J.S., Wilcox R.A., Flies D.B., Liu D., Dong H., Sica G.L., Zhu G., Tamada K. (2001). B7-H3: A costimulatory molecule for T cell activation and IFN-gamma production. Nat. Immunol..

[3] Li G., Quan Y., Che F., Wang L. (2018). B7-H3 in tumors: Friend or foe for tumor immunity?. Cancer Chemother. Pharmacol..

[4] Hofmeyer K.A., Ray A., Zang X. (2008). The contrasting role of B7-H3. Proc. Natl. Acad. Sci. USA.

[5] Castellanos J.R., Purvis I.J., Labak C.M., Guda M.R., Tsung A.J., Velpula K.K., Asuthkar S. (2017). B7-H3 role in the immune landscape of cancer. Am. J. Clin. Exp. Immunol..

[6] Chen C., Shen Y., Qu Q.X., Chen X.Q., Zhang X.G., Huang J.A. (2013). Induced expression of B7-H3 on the lung cancer cells and macrophages suppresses T-cell mediating anti-tumor immune response. Exp. Cell. Res..

[7] Vigdorovich V., Ramagopal U.A., Lazar-Molnar E., Sylvestre E., Lee J.S., Hofmeyer K.A., Zang X., Nathenson S.G., Almo S.C. (2013). Structure and T cell inhibition properties of B7 family member, B7-H3. Structure.

[8] Picarda E., Ohaegbulam K.C., Zang X. (2016). Molecular Pathways: Targeting B7-H3 (CD276) for Human Cancer Immunotherapy. Clin. Cancer Res..

[9] Du H., Hirabayashi K., Ahn S., Kren N.P., Montgomery S.A., Wang X., Tiruthani K., Mirlekar B., Michaud D., Greene K. (2019). Antitumor Responses in the Absence of Toxicity in Solid Tumors by Targeting B7-H3 via Chimeric Antigen Receptor T Cells. Cancer Cell.

[10] Fauci J.M., Straughn J.M., Ferrone S., Buchsbaum D.J. (2012). A review of B7-H3 and B7-H4 immune molecules and their role in ovarian cancer. Gynecol. Oncol..

[11] Zang X., Sullivan P.S., Soslow R.A., Waitz R., Reuter V.E., Wilton A., Thaler H.T., Arul M., Slovin S.F., Wei J. (2010). Tumor associated endothelial expression of B7-H3 predicts survival in ovarian carcinomas. Mod. Pathol..

[12] Dong P., Xiong Y., Yue J., Hanley S.J.B., Watari H. (2018). B7H3 As a Promoter of Metastasis and Promising Therapeutic Target. Front. Oncol..

[13] May M.B., Glode A. (2016). Blinatumomab: A novel, bispecific, T-cell engaging antibody. Am. J. Health Syst. Pharm..

[14] Barrett D.M., Teachey D.T., Grupp S.A. (2014). Toxicity management for patients receiving novel T-cell engaging therapies. Curr. Opin. Pediatr..

[15] Gleason M.K., Ross J.A., Warlick E.D., Lund T.C., Verneris M.R., Wiernik A., Spellman S., Haagenson M.D., Lenvik A.J., Litzow M.R. (2014). CD16xCD33 bispecific killer cell engager (BiKE) activates NK cells against primary MDS and MDSC CD33+ targets. Blood.

[16] Alderson K.L., Sondel P.M. (2011). Clinical cancer therapy by NK cells via antibody-dependent cell-mediated cytotoxicity. J. Biomed. Biotechnol..

[17] Schmohl J.U., Gleason M.K., Dougherty P.R., Miller J.S., Vallera D.A. (2015). Heterodimeric Bispecific Single Chain Variable Fragments (scFv) Killer Engagers (BiKEs) Enhance NK-cell Activity Against CD133+ Colorectal Cancer Cells. Target. Oncol..

[18] Felices M., Kodal B., Hinderlie P., Kaminski M.F., Cooley S., Weisdorf D.J., Vallera D.A., Miller J.S., Bachanova V. (2019). Novel CD19-targeted TriKE restores NK cell function and proliferative capacity in CLL. Blood Adv..

[19] Schmohl J.U., Felices M., Oh F., Lenvik A.J., Lebeau A.M., Panyam J., Miller J.S., Vallera D.A. (2017). Engineering of Anti-CD133 Tri-Specific Molecule Capable of Inducing NK Expansion and Driving Antibody-Dependent Cell-Mediated Cytotoxicity (ADCC). Cancer Res. Treat..

[20] Schmohl J.U., Felices M., Taras E., Miller J.S., Vallera D.A. (2016). Enhanced ADCC and NK Cell Activation of an Anticarcinoma Bispecific Antibody by Genetic Insertion of a Modified IL-15 Cross-linker. Mol. Ther..

[21] Schmohl J.U., Felices M., Todhunter D., Taras E., Miller J.S., Vallera D.A. (2016). Tetraspecific scFv construct provides NK cell mediated ADCC and self-sustaining stimuli via insertion of IL-15 as a cross-linker. Oncotarget.

[22] Vallera D.A., Felices M., McElmurry R., McCullar V., Zhou X., Schmohl J.U., Zhang B., Lenvik A.J., Panoskaltsis-Mortari A., Verneris M.R. (2016). IL15 Trispecific Killer Engagers (TriKE) Make Natural Killer Cells Specific to CD33+ Targets While Also Inducing Persistence, In Vivo Expansion, and Enhanced Function. Clin. Cancer Res..

[23] Felices M., Lenvik T.R., Kodal B., Lenvik A.J., Hinderlie P., Bendzick L.E., Schirm D.K., Kaminski M.F., McElmurry R.T., Geller M.A. (2020). Potent Cytolytic Activity and Specific IL15 Delivery in a Second-Generation Trispecific Killer Engager. Cancer Immunol. Res..

[24] Carson W.E., Giri J.G., Lindemann M.J., Linett M.L., Ahdieh M., Paxton R., Anderson D., Eisenmann J., Grabstein K., Caligiuri M.A. (1994). Interleukin (IL) 15 is a novel cytokine that activates human natural killer cells via components of the IL-2 receptor. J. Exp. Med..

[25] Behar G., Siberil S., Groulet A., Chames P., Pugniere M., Boix C., Sautes-Fridman C., Teillaud J.L., Baty D. (2008). Isolation and characterization of anti-FcgammaRIII (CD16) llama single-domain antibodies that activate natural killer cells. Protein Eng. Des. Sel..

[26] Vincke C., Loris R., Saerens D., Martinez-Rodriguez S., Muyldermans S., Conrath K. (2009). General strategy to humanize a camelid single-domain antibody and identification of a universal humanized nanobody scaffold. J. Biol. Chem..

[27] Fauci J.M., Sabbatino F., Wang Y., Londono-Joshi A.I., Straughn J.M., Landen C.N., Ferrone S., Buchsbaum D.J. (2014). Monoclonal antibody-based immunotherapy of ovarian cancer: Targeting ovarian cancer cells with the B7-H3-specific mAb 376.96. Gynecol. Oncol..

[28] Felices M., Chu S., Kodal B., Bendzick L., Ryan C., Lenvik A.J., Boylan K.L., Wong H.C., Skubitz A.P., Miller J.S. (2017). IL-15 super-agonist (ALT-803) enhances natural killer (NK) cell function against ovarian cancer. Gynecol. Oncol..

[29] Rhode P.R., Egan J.O., Xu W., Hong H., Webb G.M., Chen X., Liu B., Zhu X., Wen J., You L. (2016). Comparison of the Superagonist Complex, ALT-803, to IL15 as Cancer Immunotherapeutics in Animal Models. Cancer Immunol. Res..

[30] Uppendahl L.D., Felices M., Bendzick L., Ryan C., Kodal B., Hinderlie P., Boylan K.L.M., Skubitz A.P.N., Miller J.S., Geller M.A. (2019). Cytokine-induced memory-like natural killer cells have enhanced function, proliferation, and in vivo expansion against ovarian cancer cells. Gynecol. Oncol..

[31] Loo D., Alderson R.F., Chen F.Z., Huang L., Zhang W., Gorlatov S., Burke S., Ciccarone V., Li H., Yang Y. (2012). Development of an Fc-enhanced anti-B7-H3 monoclonal antibody with potent antitumor activity. Clin. Cancer Res..

[32] Kramer K., Kushner B.H., Modak S., Pandit-Taskar N., Smith-Jones P., Zanzonico P., Humm J.L., Xu H., Wolden S.L., Souweidane M.M. (2010). Compartmental intrathecal radioimmunotherapy: Results for treatment for metastatic CNS neuroblastoma. J. Neurooncol..

[33] Kramer K., Smith M., Souweidane M.M. (2014). Safety profile of long-term intraventricular access devices in pediatric patients receiving radioimmunotherapy for central nervous system malignancies. Pediatr. Blood Cancer.

[34] Souweidane M.M., Kramer K., Pandit-Taskar N., Zhou Z., Haque S., Zanzonico P., Carrasquillo J.A., Lyashchenko S.K., Thakur S.B., Donzelli M. (2018). Convection-enhanced delivery for diffuse intrinsic pontine glioma: A single-centre, dose-escalation, phase 1 trial. Lancet Oncol..

[35] Seaman S., Zhu Z., Saha S., Zhang X.M., Yang M.Y., Hilton M.B., Morris K., Szot C., Morris H., Swing D.A. (2017). Eradication of Tumors through Simultaneous Ablation of CD276/B7-H3-Positive Tumor Cells and Tumor Vasculature. Cancer Cell.

[36] Benzon B., Zhao S.G., Haffner M.C., Takhar M., Erho N., Yousefi K., Hurley P., Bishop J.L., Tosoian J., Ghabili K. (2017). Correlation of B7-H3 with androgen receptor, immune pathways and poor outcome in prostate cancer: An expression-based analysis. Prostate Cancer Prostatic Dis..

[37] Inamura K., Yokouchi Y., Kobayashi M., Sakakibara R., Ninomiya H., Subat S., Nagano H., Nomura K., Okumura S., Shibutani T. (2017). Tumor B7-H3 (CD276) expression and smoking history in relation to lung adenocarcinoma prognosis. Lung Cancer.

[38] Loos M., Hedderich D.M., Friess H., Kleeff J. (2010). B7-h3 and its role in antitumor immunity. Clin. Dev. Immunol..

[39] Yamato I., Sho M., Nomi T., Akahori T., Shimada K., Hotta K., Kanehiro H., Konishi N., Yagita H., Nakajima Y. (2009). Clinical importance of B7-H3 expression in human pancreatic cancer. Br. J. Cancer.

[40] Cooley S., He F., Bachanova V., Vercellotti G.M., DeFor T.E., Curtsinger J.M., Robertson P., Grzywacz B., Conlon K.C., Waldmann T.A. (2019). First-in-human trial of rhIL-15 and haploidentical natural killer cell therapy for advanced acute myeloid leukemia. Blood Adv..

[41] Dubois S., Conlon K.C., Muller J.R., Hsu-Albert J., Beltran N., Bryant B.R., Waldmann T.A. (2017). IL15 Infusion of Cancer Patients Expands the Subpopulation of Cytotoxic CD56(bright) NK Cells and Increases NK-Cell Cytokine Release Capabilities. Cancer Immunol. Res..

[42] Miller J.S., Morishima C., McNeel D.G., Patel M.R., Kohrt H.E.K., Thompson J.A., Sondel P.M., Wakelee H.A., Disis M.L., Kaiser J.C. (2018). A First-in-Human Phase I Study of Subcutaneous Outpatient Recombinant Human IL15 (rhIL15) in Adults with Advanced Solid Tumors. Clin. Cancer Res..

[43] Romee R., Cooley S., Berrien-Elliott M.M., Westervelt P., Verneris M.R., Wagner J.E., Weisdorf D.J., Blazar B.R., Ustun C., DeFor T.E. (2018). First-in-human phase 1 clinical study of the IL-15 superagonist complex ALT-803 to treat relapse after transplantation. Blood.

[44] Sarhan D., Brandt L., Felices M., Guldevall K., Lenvik T., Hinderlie P., Curtsinger J., Warlick E., Spellman S.R., Blazar B.R. (2018). 161533 TriKE stimulates NK-cell function to overcome myeloid-derived suppressor cells in MDS. Blood Adv..

[45] Felices M., Lenvik A.J., McElmurry R., Chu S., Hinderlie P., Bendzick L., Geller M.A., Tolar J., Blazar B.R., Miller J.S. (2018). Continuous treatment with IL-15 exhausts human NK cells via a metabolic defect. JCI Insight.

[46] Bi J., Tian Z. (2017). NK Cell Exhaustion. Front Immunol..

[47] Romee R., Foley B., Lenvik T., Wang Y., Zhang B., Ankarlo D., Luo X., Cooley S., Verneris M., Walcheck B. (2013). NK cell CD16 surface expression and function is regulated by a disintegrin and metalloprotease-17 (ADAM17). Blood.

[48] Belisle J.A., Gubbels J.A., Raphael C.A., Migneault M., Rancourt C., Connor J.P., Patankar M.S. (2007). Peritoneal natural killer cells from epithelial ovarian cancer patients show an altered phenotype and bind to the tumour marker MUC16 (CA125). Immunology.

[49] Watanabe M., Kono K., Kawaguchi Y., Mizukami Y., Mimura K., Maruyama T., Izawa S., Fujii H. (2010). NK cell dysfunction with down-regulated CD16 and up-regulated CD56 molecules in patients with esophageal squamous cell carcinoma. Dis. Esophagus.

[50] Petricevic B., Laengle J., Singer J., Sachet M., Fazekas J., Steger G., Bartsch R., Jensen-Jarolim E., Bergmann M. (2013). Trastuzumab mediates antibody-dependent cell-mediated cytotoxicity and phagocytosis to the same extent in both adjuvant and metastatic HER2/neu breast cancer patients. J. Transl. Med..

[51] Rossello A., Nuti E., Ferrini S., Fabbi M. (2016). Targeting ADAM17 Sheddase Activity in Cancer. Curr. Drug Targets.

[52] Jing Y., Ni Z., Wu J., Higgins L., Markowski T.W., Kaufman D.S., Walcheck B. (2015). Identification of an ADAM17 cleavage region in human CD16 (FcgammaRIII) and the engineering of a non-cleavable version of the receptor in NK cells. PLoS ONE.

[53] Zhu H., Blum R.H., Bjordahl R., Gaidarova S., Rogers P., Lee T.T., Abujarour R., Bonello G.B., Wu J., Tsai P.F. (2020). Pluripotent stem cell-derived NK cells with high-affinity noncleavable CD16a mediate improved antitumor activity. Blood.

