# Supplementary material for: NK-Cell-Mediated Targeting of Various Solid Tumors Using a B7-H3 Tri-Specific Killer Engager In Vitro and In Vivo
Source: Cancers (Basel). 2020 Sep 18;12(9):2659. doi: 10.3390/cancers12092659 (PMC7564091; doi:10.3390/cancers12092659)
Supplement: Supplementary file 1 [file cancers-12-02659-s001.pdf]

*cancers*

*Supplementary material*

# NK-Cell-Mediated Targeting of Various Solid Tumors Using a B7-H3 Tri-Specific Killer Engager In Vitro and In Vivo

**Daniel A. Vallera, Soldano Ferrone, Behiye Kodal, Peter Hinderlie, Laura Bendzick, Brianna Ettestad, Caroline Hallstrom, Nicholas A. Zorko, Arpit Rao, Naomi Fujioka, Charles J. Ryan, Melissa A. Geller, Jeffrey S. Miller, and Martin Felices**

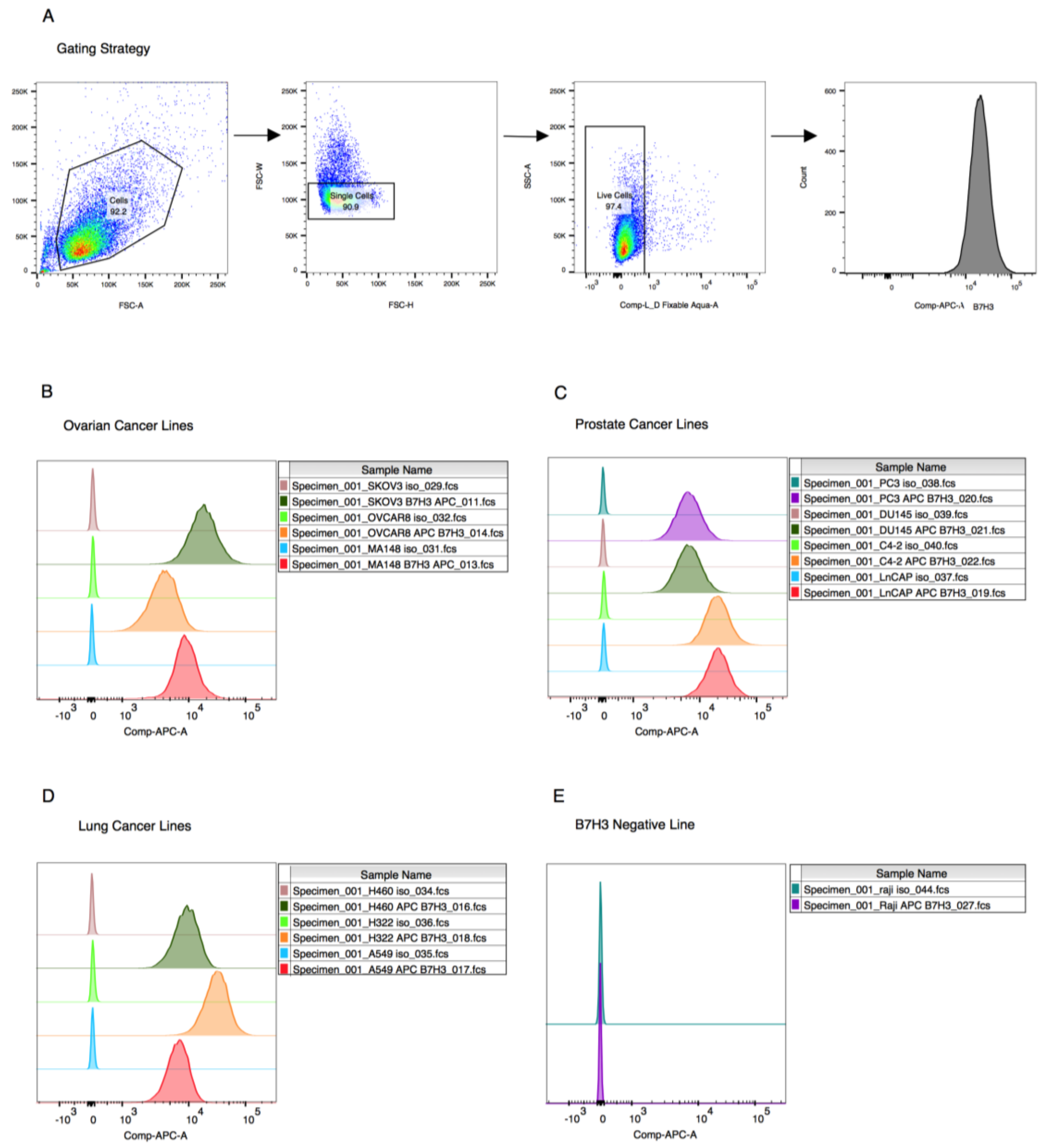


**Figure 1.** To evaluate expression of B7-H3 on a broad spectrum of cancer cell lines, an APC labeled anti-B7-H3 antibody (clone 7-517, Invitrogen) was used. (**A**) Gating strategy for flow cytometric evaluation of B7-H3 on tumor cell lines. B7-H3 expression on (**B**) ovarian cancer cells, (**C**) prostate cancer cells, (**D**) lung cancer cells, and (**E**) lymphoma cells (as a negative control).

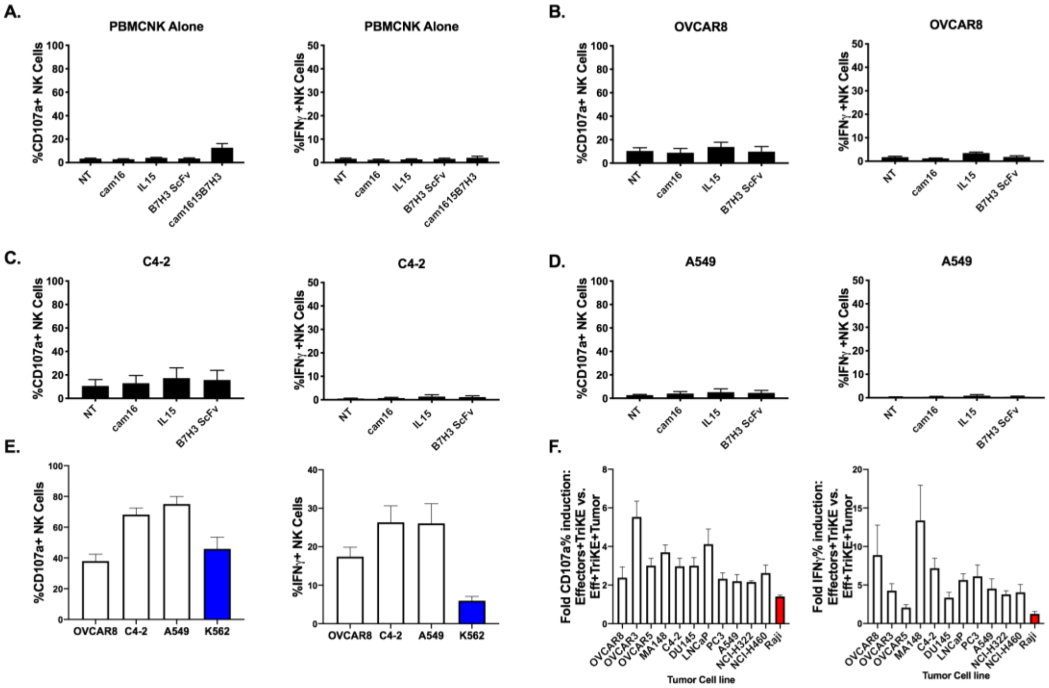


**Figure S2.** (**A**) Pooled analysis of proportion of NK cells from healthy donors expressing CD107a+ (left) or IFNγ (right) when incubated with noted treatments and no targets, indicative of basal NK cell activity (N = 8). (**B**) OVCAR8, (**C**) C4-2, and (**D**) A549 targets were incubated with PBMCs and individual components of TriKE (cam16, IL-15 and B7-H3 scFv), in comparison to no treatment (NT), to determine the level of degranulation (CD107a+) and IFNγ production induced on NK cells with these individual components (N = 3). (**E**) NK cells were incubated with 30 nM TriKE and OVCAR8 (N = 7), C4-2 (N = 9), A549 (N = 5) cells to assess ADCC or were incubated with K562 targets (in blue, N = 11) to determine activation via natural cytotoxicity and degranulation (CD107a, left) and intracellular IFNγ production was determined by flow cytometry. (**F**) Fold activation was calculated for a number of B7-H3 expressing tumor lines, and a B7-H3 negative line (Raji cells in red), to determine to determine functional specificity of the cam1615B7H3 TriKE. The fold activation calculation takes into account background activation by the TriKE alone (in the absence of targets) by dividing the proportion of NK cells expressing CD107a (left) or IFNγ (right) on PBMC+noted tumor+TriKE by the PBMC+TriKE alone (N = 4–12).

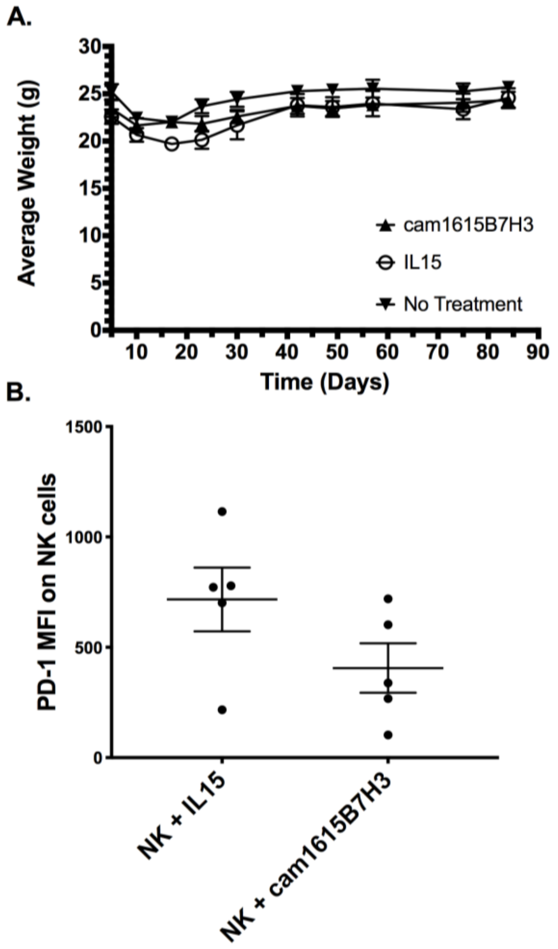


**Figure S3.** (**A**) NSG mice were irradiated (180 cGy), to enhance NK cell engraftment, and injected with 1 million NK cells 3 days later. Mice were then either left untreated, or treated with 5 ug/injection IL15 (3 days a week; MWF), or 30 ug/injection cam1615B7H3 (5 days a week; MTWThF) for three weeks. To elucidate toxicity, weights were tracked over the course of 90 days, starting at the time of treatment (N = 5). (**B**) Xenogeneic ovarian cancer MA-148-Luc model was carried out with injected NK cells and noted treatments. 21 days after initial treatment mice were harvested and peritoneal lavages were carried out to evaluate PD-1 expression on CD56+CD3− NK cells by flow cytometry. Scatter plots shown displaying PD-1 median fluorescence intensity on NK cells.

© 2020 by the authors. Licensee MDPI, Basel, Switzerland. This article is an open access article distributed under the terms and conditions of the Creative Commons Attribution (CC BY) license (http://creativecommons.org/licenses/by/4.0/).
